# Transcranial Magnetic Stimulation as a Diagnostic and Therapeutic Tool in Various Types of Dementia

**DOI:** 10.3390/jcm10132875

**Published:** 2021-06-28

**Authors:** Jakub Antczak, Gabriela Rusin, Agnieszka Słowik

**Affiliations:** Department of Neurology, Jagiellonian University Medical College, Jakubowskiego 2, 30-688 Krakow, Poland; jakub.antczak@uj.edu.pl (J.A.); slowik@neuro.cm-uj.krakow.pl (A.S.)

**Keywords:** dementia, Alzheimer’s disease, vascular dementia, dementia with Lewy bodies, frontotemporal dementia, Parkinson’s disease, mild cognitive impairment, transcranial magnetic stimulation, paired pulse transcranial magnetic stimulation, biomarker

## Abstract

Dementia is recognized as a healthcare and social burden and remains challenging in terms of proper diagnosis and treatment. Transcranial magnetic stimulation (TMS) is a diagnostic and therapeutic tool in various neurological diseases that noninvasively investigates cortical excitability and connectivity and can induce brain plasticity. This article reviews findings on TMS in common dementia types as well as therapeutic results. Alzheimer’s disease (AD) is characterized by increased cortical excitability and reduced cortical inhibition, especially as mediated by cholinergic neurons and as documented by impairment of short latency inhibition (SAI). In vascular dementia, excitability is also increased. SAI may have various outcomes, which probably reflects its frequent overlap with AD. Dementia with Lewy bodies (DLB) is associated with SAI decrease. Motor cortical excitability is usually normal, reflecting the lack of corticospinal tract involvement. DLB and other dementia types are also characterized by impairment of short interval intracortical inhibition. In frontotemporal dementia, cortical excitability is increased, but SAI is normal. Repetitive transcranial magnetic stimulation has the potential to improve cognitive function. It has been extensively studied in AD, showing promising results after multisite stimulation. TMS with electroencephalography recording opens new possibilities for improving diagnostic accuracy; however, more studies are needed to support the existing data.

## 1. Introduction

With life expectancy still increasing, dementia has become one of the main healthcare and social burdens in developed countries. In 2015, it affected nearly 50 million people, and it will affect over 130 million in 2050 [1]. Global costs of dementia are estimated at USD 1 trillion [2]. Dementia is the main cause of dependence and disability among older people and makes a profound impact on the life of their relatives [3]. Among about 50 identified types and etiologies of dementia, the most prevailing ones are Alzheimer’s disease (AD), vascular dementia (VAD) and dementia with Lewy bodies (DLB). Less frequent, but still with an impact on public health, are frontotemporal dementia (FTD) and Parkinson’s disease (PD) with dementia (PDD) [4,5,6,7]. A significant population fulfills the criteria of mild cognitive impairment (MCI)—a condition characterized by decline of cognitive performance not interfering with activities of daily living, but more advanced than in healthy peers and associated with the risk of developing dementia [8]. The definite diagnosis of dementia is based on histopathological changes seen in an autopsy [9,10,11]. In most cases, diagnosis is made upon initial and prevailing signs and symptoms, age of onset, family history and other demographic and clinical data. Despite the fact that the clinical picture of various degenerative and other dementia types has been extensively described, it is still a challenge to detect the proper etiology in vivo and even to differentiate the pathological process from normal aging. The symptoms may be subtle and overlapping between different types, which often results in the diagnosis of mixed dementia [12]. As a result, a number of radiologic, laboratory and neurophysiologic techniques have been involved to increase the reliability of in vivo diagnosis as well as to create screening tests. Since about twenty years ago, a number of studies have reported promising results regarding the use of transcranial magnetic stimulation (TMS) as an adjuvant diagnostic tool enabling assessment of the excitatory and inhibitory properties of the motor cortex as well as brain connectivity. Paired pulse TMS (ppTMS) is a TMS modality that may provide insight into the neurophysiological properties of particular dementia subtypes and MCI and enable the monitoring of disease progression [13,14,15,16,17]. Moreover, through its potential to induce brain plasticity, TMS is now also recognized as an alternative therapeutic option for various neuropsychiatric conditions including dementia. The therapeutic modality of TMS is termed repetitive TMS (rTMS). In this method, series of magnetic stimuli produce long-lasting changes in local cortical excitability, which—depending on site, pattern and intensity of stimulation—may be associated with cognitive enhancement, mood stabilization and other beneficial, therapeutic effects [18,19,20].

### 1.1. TMS

The method uses brief pulses of a time-varying magnetic field with intensity up to around 1.5 T, which is generated by the stimulator and attached coil held over the scalp area selected by the TMS operator. The magnetic field is able to penetrate into the cortex and depolarize the neurons underneath the coil. Structures located deeper and beside the coil are not excited directly, as the magnetic field decreases exponentially with the distance from the coil. However, they can be influenced indirectly via neuronal networks [21]. At the beginning of TMS, which was in 1985, single pulses were used to excite the primary motor cortex (PMC) and, in turn, to induce descending volleys down the pyramidal tract and the peripheral motor pathways and finally, to induce the contraction of skeletal muscles, which was recorded electrophysiologically as the motor evoked potential (MEP). This basic technique was used and still is, primarily in neurology, to assess the conduction in central motor pathways [22]. In the case of diagnostics and research on dementia, stimulation with single pulses may contribute to the assessment of motor cortical excitability and inhibition by estimating the motor threshold (MT) and the cortical silent period (CSP). MT is defined as the minimal intensity of a magnetic field able to reliably evoke MEPs. Estimation of MT is operationalized in most studies, as the lowest intensity of the magnetic field—expressed as the percentage of the maximal stimulator output—evokes MEPs of amplitudes over 50 µV after at least five out of ten stimuli [23]. CSP is the period of involuntary relaxation of skeletal muscles, which follows the MEP, when TMS is applied during the voluntary efforts of those muscles. When examined according to the guidelines published by the International Federation of Clinical Neurophysiology (IFCN) [23], CSP lasts between 100 and 200 milliseconds [ms] and represents cortical and spinal inhibition, partly generated by intracortical GABA_B_ circuits and in part by spinal mechanisms [24].

### 1.2. Paired Pulse TMS

ppTMS gives a more detailed and dimensional insight into excitability and inhibition of the motor cortex. In this method, the magnetic stimulus elicited to evoke motor response is preceded by a conditioning stimulus. This conditioning stimulus may be elicited by the same magnetic coil and in that case, its strength is usually below MT. In addition, it may be an electric stimulus applied to excite the peripheral nerve. A test stimulus is second, and its strength is adjusted to evoke motor responses of a certain amplitude, e.g., 0.2 mV, when applied without the conditioning stimulus. Depending on the length of the interval, the conditioning stimulus may have an inhibitory effect, i.e., it may decrease the amplitude of the MEP evoked by the test stimulus, or it may have the opposite effect [25]. In the method called the threshold tracking technique, which is gaining interest thanks to reduced variability of its results, paired pulses are elicited repetitively, every few seconds. The built-in algorithm automatically adjusts the strength of the test stimuli, keeping the amplitude of the MEP response unchanged with respect to the responses after the test stimulus alone. Next, the cortical inhibition and facilitation by the conditioning stimulus are expressed in threshold tracking as the increase and decrease in the strength of the test stimulus, respectively [26]. The parameters obtained with ppTMS described below are, meanwhile, the main established measurements of cortical excitability. 

### 1.3. Short-Interval Cortical Inhibition

Short-interval cortical inhibition (SICI) occurs when the test stimulus is elicited 1 to 4 ms after the conditioning stimulus, and the inhibition can induce up to a 40% decrease in the MEP amplitude (or increase in the stimulus strength in the threshold tracking technique). The circuits within the motor cortex, consisting primarily of GABA_A_ neurons, are thought to be responsible for SICI [27]. 

### 1.4. Intracortical Facilitation

For intracortical facilitation (ICF), the interstimulus interval (ISI) is between 7 and 20 ms, and the amplitude of MEP may even quadruple [28,29]. The mechanism of ICF is not clearly elucidated. ICF is reduced by the administration of the NMDA receptor antagonist dextromethorphan [30]. Hence, it is likely to be mediated by glutamatergic transmission.

### 1.5. Long-Interval Intracortical Inhibition

Long-interval intracortical inhibition (LICI) requires a suprathreshold conditioning stimulus and ISI between 50 and 300 ms [31,32,33]. It is mediated predominantly by GABA_B_ receptors [34,35].

### 1.6. Short-Latency Afferent Inhibition

In short-latency afferent inhibition (SAI), the test stimulus is preceded by the electric stimulus to the contralateral, peripheral nerve, usually the median nerve, at the wrist. ISI depends on the latency of the responses recorded over the primary sensory cortex as somatosensory-evoked potential after electric stimulation on the same site as the conditioning stimulus. Usually, the latency is around 20 ms, and the respective ISI is 22 ms. Two milliseconds are added, as that is the time duration needed for the transmission via sensorimotor projections. In normal subjects, the conditioning stimuli should inhibit the test responses [36]. 

### 1.7. Repetitive Transcranial Magnetic Stimulation

In rTMS, series of magnetic stimuli are applied to repetitively depolarize targeted neurons. Repetitive depolarization induces synaptic plasticity, which outlasts the stimulation. Depending on the stimulation pattern, induced plasticity may be directed toward long term potentiation (LTP) with the enhancement of synaptic transmission and increased neuronal firing and metabolism or long-term depression (LTD) with opposite changes. rTMS with high frequencies, i.e., five or more stimuli per second, and a more complex pattern called intermittent theta burst stimulation (iTBS), as well as several other less frequently used patterns, induce predominantly LTP, whereas low-frequency stimulation (one stimulus or less per second), continuous theta burst stimulation (cTBS) and several other protocols result predominantly in LTD [37]. One of the well-known neurophysiologic manifestations of induced plasticity is the increase in the MEP amplitude after high frequency stimulation [38]. A clinical correlate is the increase in the muscle strength or dexterity and reduction of spasticity in post-stroke patients and in other neurologic conditions [39,40,41,42,43,44]. Stimulation over nonmotor cortical areas will induce other effects, which may also bring a therapeutic benefit [19]. While the effects of one rTMS session, consisting typically of several hundred to several thousand stimuli, last for minutes, hours or rarely, days, repeated sessions may induce long-lasting effects, bringing significant alterations to the severity of psychiatric or neurologic disease and improving quality of life [19].

## 2. Materials and Methods

We conducted this review following the Scale for the Assessment of Narrative Review Articles items [45]. We included clinical studies regarding: (1) the use of TMS parameters as dementia biomarkers and (2) the use of repetitive TMS in the treatment of dementia. We searched the electronic databases of MEDLINE through PubMed (United States National Library of Medicine, Bethesda, MD, USA), Scopus (Elsevier, Amsterdam, Netherlands) and Google Scholar (Google, Mountain View, CA, USA).for relevant research articles published up to March 2021. The search strategy consisted of the following terms used in combination: “transcranial magnetic stimulation”, “TMS”, “dementia”, “cognitive impairment”, “Alzheimer’s disease”, “dementia with Lewy bodies”, “frontotemporal dementia”, “vascular dementia”, “Parkinson’s disease”, “mild cognitive impairment”, “diagnostics”, “diagnosis”, “therapy”, “treatment”, “biomarker”. We modified the search strategy for other methods (MeSH or free text terms), as appropriate. We conducted additional manual searches of the references of the related articles in order to gather information about the relevant supporting literature.

In the following sections, we present the diagnostic and therapeutic utility of TMS in particular types of dementia. 

## 3. Alzheimer’s Disease

Alzheimer’s disease (AD) is the most common type of dementia, affecting about 3.9% of people over 60 years of age [46]. Distinctive clinical features of AD include short-term memory loss, speech deficits, lack of motivation and self-care and prominent changes in behavior and mood. In late stages, patients reveal motor disturbances such as rigidity and hypokinesia. Extracellular amyloid beta-protein deposition with intracellular clusters of hyperphosphorylated tau protein are the neuropathological hallmarks of AD. These changes primarily affect cholinergic transmission [47]. 

### 3.1. Increased Cortical Excitability in AD

The first data, which were obtained with single pulse TMS, revealed a decrease of MT [48,49] as a marker of hyperexcitability in AD patients, which was confirmed in further research [50]. It is observed even in the early stages of AD, long before the onset of the motor symptoms [51], and probably reflects the presence of AD-specific pathologic changes in the primary motor cortex [52]. In advanced stages of AD, MT increases due to marked cortical atrophy and the decrease in density of cortico-spinal tract fibers [53,54].

### 3.2. Impaired Cortical Plasticity

An increase in the amplitude of MEP after sessions of rTMS, which occurs in healthy subjects, does not occur in AD or is significantly limited [55,56]. Interestingly, the other direction of cortical plasticity, i.e., LTD after low frequency rTMS, is probably significantly better preserved [57].

### 3.3. Impaired Cortical Inhibition

Studies have consistently revealed the impairment of SAI in AD [58,59,60,61,62,63,64,65]. Reduction of SICI and LICI was reported less consistently [49,58,66,67,68,69,70]. However, together with MT, all three parameters showed significant differences in comparison to healthy controls in a recent meta-analysis [71]. ICF, on the other hand, was not altered [71]. Acetylcholinesterase inhibitors used to delay AD progression enhance cortical inhibition, expressed by SAI and SICI [60,72,73], with no impact on excitability (MT) [72,73]. 

### 3.4. Diagnostic Sensitivity and Specificity

According to a meta-analysis by Mimura et al. [71], the effect size of MT and SAI decrease in AD is large, and it is medium for the decrease of SICI. Recently, Benussi et al. [65] conducted a multicenter study in order to examine the classification performance of TMS outcome measures in the differential diagnosis of three neurodegenerative dementias. They included 273 patients diagnosed as AD, 67 as DLB and 207 as FTD. Measurements included MT, SICI, LICI, ICF and SAI. The machine learning model was employed to make a diagnosis based upon the derived parameters. The classification accuracy yielded was very high (ranging from 0.89 to 0.92) as were the precision (0.86–0.92) and other parameters of overall diagnostic accuracy. Another study investigated TMS as an adjuvant diagnostic tool to routine clinical assessment [63]. TMS increased diagnostic accuracy from 82% (clinical work-up alone) to 98%, while PET and cerebrospinal fluid-based markers of amyloidosis increased the accuracy to 99%.

### 3.5. Therapy with rTMS

Among dementia types, the therapeutic potential of rTMS has been investigated most extensively in AD. In most reports, the DLPFC, uni- or bilaterally, or the precuneus were selected as the target for high frequency (between 10 and 20 Hz) stimulation [74,75,76]. In the majority of studies, a beneficial effect was documented in various neurocognitive testing such as the AD Assessment Scale-cognitive subscale (ADAS-cog), the Mini-Mental State Examination (MMSE) and the Montreal Cognitive Assessment (MoCA). The studies were, however, small and, in many cases, uncontrolled. 

Among trials with rTMS, the study of Lee at al. [77] gained special attention, as the therapeutic effect exceeded that of acetylcholinesterase inhibitors. The study was among the few that used a multi-site rTMS approach. Stimulation of both dorsolateral prefrontal and parietal somatosensory-associated cortices as well as Broca’s and Wernicke’s areas was interleaved with specifically sequenced cognitive training. A similar approach was investigated in a recent multicenter study, which recruited over 130 patients with mild to moderate AD [78]. Thirty daily sessions of 10 Hz rTMS over six areas (targeted with help of the neuronavigation system) were combined with cognitive training, building up the therapeutic system known as neuroAD™ (Neuronix Ltd., Israel). The results showed improvement in ADAS-cog that lasted over a month after the therapy and which was not present in the sham group. Another study using neuroAD™ Therapy System confirmed the beneficial effect on cognition and also noted the correlation of the therapeutic effect with preservation of cortical plasticity in the course of AD [79]. The multisite approach is, to date, the only rTMS procedure for dementias which has obtained therapeutic recommendation of IFCN [19].

One has to bear in mind that the patients recruited to studies with TMS were diagnosed in vivo and therefore, some heterogeneity within the treated groups cannot be excluded. Moreover, while the majority of the studies involved subjects diagnosed with possible/probable AD according to the National Institute of Neurological and Communicative Disorders and Stroke–Alzheimer’s Disease and Related Disorders Association (NINCDS-ADRDA) [48,49,50,51,54,56,57,58,60,61,62,67,68,69,70,72,79], only few used its revised version and recommendations the from National Institute on Aging-Alzheimer’s Association (NIA-AA) incorporating biomarkers (including [63,65,75,79]) [80,81,82]. These factors may potentially compromise diagnostic reliability and the therapeutic efficacy of rTMS in AD, and the need for long-term, longitudinal studies including autopsy reports is emerging as the use of TMS in patients with dementia increases. The incorporation of TMS into diagnostic and therapeutic workups, although warranted by published results, must be done with caution and in the proper context of clinical data and other diagnostic and therapeutic options.

## 4. Vascular Dementia

Vascular dementia is the second most common type of dementia and is defined as cognitive dysfunction where a cerebrovascular or cardiovascular disease is a causative or contributing factor. Vascular cognitive impairment is a broad term encompassing all sorts of cognitive disorders caused by vascular brain injury or dysfunction leading to cerebral blood flow impairment [83]. Similarly to AD patients, those with VAD show reduced RMT across studies [70,84,85,86]. Only a few studies have reported the impairment of SAI [70,87], and another study has shown that SAI may be normal [88]. Such a discrepancy seems to represent the frequent overlap between those two entities [15,84]. SAI is more consistently impaired in a specific, genetic form of VAD called cerebral, autosomal-dominant arteriopathy with subcortical infarcts (CADASIL) [89], often with a decrease of ICF [90,91,92]. Interestingly, impairment of SAI cannot be reversed by dopamine in CADASIL, while it can in AD [92]. This finding reflects the differences in the mechanisms of impairment of cholinergic transmission which in CADASIL, may not be the result of neurodegenerative processes but of the specific locations of the infarcts, which interfere with the cholinergic pathways [92]. In most studies, other parameters obtained with TMS seem not to be consistently changed in VAD [13]. Therapeutic studies in humans are limited to one pilot trial by Rektorova et al. [93], where one session of high frequency rTMS over the left DLPFC improved executive functions. In contrast, a large number of studies have investigated magnetic stimulation in animal models of VAD, mainly with promising results [94,95,96,97,98,99,100].

## 5. Dementia with Lewy Bodies

DLB is a synucleinopathy affecting the subcortical structures and is regarded as the third most common type of dementia. Typical clinical features include cognitive fluctuations, decline of executive functions, parkinsonian syndrome without tremor, visual hallucinations and oversensitivity to neuroleptics. It is classified as one of the types of atypical parkinsonism along with progressive supranuclear palsy, corticobasal syndrome and others. Despite all of them having their distinctive clinical features, the differential diagnosis is still challenging. Moreover, the clinical picture of DLB at an early stage might overlap with AD. Cognitive decline in both AD and DLB may be associated with the disconnection of cortical areas from their source of cholinergic innervations of the basal forebrain (nucleus basalis of Meynert and the medial septum nuclei) and nuclei of the brainstem (pedunculopontine nucleus) [101]. Diagnostic accuracy may be improved in DLB using presynaptic dopaminergic imaging, but the method still lacks sufficient validation on neuropathological diagnosis [102].

Similarly to AD, a number of studies with TMS have reflected the impairment of cholinergic transmission by showing the decrease of SAI [62,103]. Interestingly, while a decrease of SAI was comparable between AD and DLB patients, it showed a correlation with the severity of different symptoms, i.e., with visual hallucinations in DLB and manic state in AD [62]. A study of [104], which preceded both mentioned studies, showed contradictive results, with SAI being significantly decreased only in AD, but not in DLB patients. The authors supposed that the cortico–cortical disconnection in DLB patients was less pronounced than in AD. This notion may be supported by the fact that in studies conducted on bigger samples, the difference in SAI between DLB and healthy controls has reached statistical significance [62,104,105]. Furthermore, in contrast to AD, no significant changes in MT are observed in DLB, which corresponds with the clinical lack of pyramidal signs [103,106]. 

A study using ppTMS revealed reduced SICI and ICF in DLB, cortico-basal syndrome (CBS) and PSP, but not in AD [105]. On the other hand, SAI was decreased in both AD and DLB, but not in PSP and CBS. These results characterize the pathology of DLB, which involves not only the disruption of cortical inhibition and facilitation mediated by GABA_B_ and glutaminergic circuits, but also impairment of cholinergic transmission. In addition, the study documented the diagnostic potential of multimodal TMS measurements: The authors used neurophysiological outcome measures to construct a diagnostic decision tree model in order to correctly allocate patients with studied types of atypical parkinsonism and healthy controls. The results yielded a diagnostic accuracy of 85.2% for DLB subjects. A unique finding among dementias was the correlation between phosphene threshold and the severity of visual hallucinations [107]. Phosphenes are the visual hallucinations that are experienced after TMS over the occipital area. The study, however, did not find differences in the phosphene threshold between patients and healthy controls, and the relevance of the documented correlation has not been investigated in further studies. 

Only one study used rTMS in DLB. In the six subjects, an improvement in depression was seen [108]. The influence of rTMS on other symptoms was not investigated. 

## 6. Frontotemporal Lobar Degeneration

Frontotemporal lobar degeneration (FLTD) is a group of degenerative disorders affecting the temporal and/or frontal lobes, consisting of primary progressive aphasia (PPA) and frontotemporal lobar dementia (FTD) with its subtypes of behavioral variant (bv-FTD), progressive non-fluent aphasia (PNFA) and semantic dementia [109]. The leading symptoms may be social behavior or personality disturbances and/or disordered speech. In a proportion of patients, signs of motor neuron impairment or extrapyramidal symptoms coexist. The accumulations of cortical tau or TDP-43 inclusions are the pathological hallmarks of FTLD [110].

Single-pulse TMS studies in FTD revealed impairment of the corticospinal tract such as reduced or absent MEP or prolonged central motor conduction time in a significantly larger proportion of patients than those presenting clinical signs from upper motor neurons [72,111]. This finding seemed to be especially robust among patients with PNFA [111]. ppTMS studies have shown impaired glutamatergic and GABAergic neurotransmission. The aforementioned, multicenter study and other studies have documented the reduction of ICF, SICI and LICI with preservation of SAI [72,112,113]. 

Recently, a group of patients with FTD underwent iTBS and cTBS of primary motor areas to explore cortical plasticity. Interestingly, the response to both stimulations (increase and decrease of MEP amplitude, respectively) was decreased only among patients presenting extrapyramidal symptoms [114]. In another recent study, TMS was successfully used to monitor a therapeutic trial for FTD with palmitoylethanolamide combined with luteoline. Beneficial cognitive effects, mediated probably by anti-inflammatory and neuroprotective mechanisms, were seen in the Neuropsychiatric Inventory (NPI) and Frontal Assessment Battery (FAB). Concurrently, the restoration of LICI and the enhancement of TMS-induced, high-frequency oscillations were registered [115]. Finally, some improvement in the results of the Montreal cognitive assessment and other cognitive tests was seen in FTD after high frequency rTMS over bilateral prefrontal areas in a small, open study [116].

## 7. Parkinson’s Disease with Dementia

In PD, basal ganglia degeneration may lead to cognitive decline resulting in PDD, which mainly involves deficits in executive functions with relatively preserved memory, learning and higher-level language abilities. As documented with neuropathological and neuroimaging data, PDD depends on the disruption of the fronto-striatal dopamine networks and cholinergic deficiency extending to frontal areas [117,118]. In accordance with these findings, Celebi et al. [119] demonstrated a significant decrease of SAI in PDD patients in comparison to healthy controls and to PD patients without dementia. The degree of SAI impairment seems to be comparable to that in AD and shows similar correlation with cognitive dysfunction. Cholinergic impairment in PDD was also reflected in the recording of CSP, which was prolonged in comparison to healthy controls [86], whereas in nondemented PD patients, it was shortened [22]. Interestingly, in another study with non-demented PD patients, the reduction of SAI showed an association with visual hallucinations as it did in DLB [120] and with other non-motor PD symptoms such as REM sleep behavior disorder, dysphagia and olfactory impairment. This finding led to the hypothesis that these symptoms might herald the onset of PDD [121]. 

A recent, randomized clinical trial assessed the effects of rTMS on cognitive impairment in PDD [122]. Real high-frequency or sham rTMS was applied over the hand area of each motor for 10 days, followed by 5 booster sessions every month for 3 months. The results showed only a minor effect on cognitive functions and a significant improvement of motor function in the active group.

## 8. Mild Cognitive Impairment

MCI is a neurocognitive disorder that includes a decline of mental processes halfway between normal aging and dementia. According to the accepted definition, a decline in cognition must be present in at least one domain, but it is not sufficient to diagnose dementia and is not significant enough to impact the instrumental activities of daily living [123]. People suffering from MCI are at risk of developing various types of dementia [124], and the characteristics of their cognitive deficits as well as radiologic and laboratory biomarkers can indicate the most probable direction of imminent conversion [125,126,127]. Changes in TMS parameters resemble those found in particular dementia types, although they are less pronounced.

As cited above, the meta-analysis of Mimura et al. [71] showed that MT, SAI, LICI and SICI were different in AD and healthy controls. In the same work, respective calculations for MCI revealed that only MT was lower than in healthy controls. SAI showed only a tendency toward reduction. LICI and SICI seemed to be similar to normal; however, data from only two studies were available. The study of Benussi et al. [17] differentiated recruited patients into MCI-Alzheimer’s disease (MCI-AD), MCI-frontotemporal dementia (MCI-FTD) and MCI-dementia with Lewy bodies (MCI-DLB). SICI and ICF were reduced in MCI-FTD and MCI-DLB with respect to healthy controls. SAI was reduced in MCI-AD and MCI-DLB and LICI was impaired in FTD, although not significantly. The authors employed the machine learning model to diagnose the MCI subtypes on the basis of TMS measurements, similarly to what they did with patients with overt dementias [65]. The prediction of particular diagnoses showed high accuracy (0.72–0.86) and high precision (0.72–0.90), which was only slightly lower than in overt dementias [65]. Overall, according to the results of the study of Benussi et al. [17], TMS could reliably diagnose MCI, which was not in full agreement with the meta-analysis of Mimura et al. [71]. It could be supposed that the methodological discrepancies between studies included in the meta-analysis and the need for more restrictive statistical comparisons might account for these discrepancies. 

Padovani et al. [64] observed impairment of SAI with unimpaired SICI and ICF among AD MCI patients in comparison to non-AD MCI. These findings suggested the existence of disturbed cholinergic pathways among the former. For differential diagnosis of AD MCI and non-AD MCI, they proposed SAI and the SICI-ICF/SAI ratio as electrocortical biomarkers with high specificity and sensitivity. This approach showed similar diagnostic accuracy in MCI patients as surrogate neuropathological hallmarks. Moreover, the addition of TMS measurements enhanced the diagnostic confidence for MCI stages of AD, FTD and DLB in comparison to clinical work-up alone and in comparison to clinical work-up and amyloid biomarkers [128]. 

rTMS has been recently investigated with respect to its procognitive effect in MCI. In most studies, the prefrontal cortex has been targeted with high frequency stimulation. A meta-analysis containing nine studies demonstrated that rTMS could yield a significant, beneficial effect on cognitive functions and memory [129]. Another meta-analysis, which included 13 studies, albeit on both MCI and AD patients, resulted in a similar conclusion. The bigger sample allowed, however, subgroup analysis, which indicated that low-frequency rTMS over the right DLPFC might improve memory, and rTMS over the inferior frontal gyrus might enhance executive performance [130]. 

The development of non-ferromagnetic electrodes and appropriate amplifiers made it possible to record the responses of electroencephalography (EEG) to TMS pulses. Moreover, the introduction of high-resolution systems containing up to 256 recording electrodes increased the spatial resolution of EEG. These advances allowed, recently, measuring excitability and connectivity beyond the motor cortex. The TMS pulse evokes the EEG response, which starts several milliseconds after the pulse and lasts up to several hundred milliseconds. Depending on the stimulus strength, the stimulation site and brain plasticity as well as connectivity this response may be recorded under various electrodes with various latencies and amplitudes. Up to now, the main findings have concerned the P30 component, which can be usually well-delineated [131]. In general, this component, recorded from the most EEG electrodes, is reduced in AD, reflecting the impaired connections between cortical areas [132]. Such impairment was also documented by functional neuroimaging, which led to coining the term “disconnection syndrome” as a description of one of the key pathological features of AD [133]. Over this background, several exceptions have been documented. One is the motor cortex, where responses seem to be stronger [134]. This finding may be attributed to the compensatory mechanism, which increases regional excitability, aiming to preserve the functional performance of the motor system. An increase in P30 amplitude was observed also in the prefrontal areas and was correlated inversely with cognitive decline in AD [135], suggesting increased, maladaptive connectivity between the prefrontal areas and other regions as another compensatory mechanism in the course of the overall decline of brain connectivity. On the other hand, some regions such as the temporo-parietal area, ipsilateral to the stimulated motor cortex or the contralateral centro-frontal cortex, may exhibit more pronounced suppression of connectivity than the others, which may result from specific patterns of neurodegeneration [136]. TMS-evoked EEG responses also showed usefulness in the monitoring of therapeutic effects: In the study of Assogna et al. [115], FTD patients were treated with palmitoylethanolamide combined with luteoline, which led to improvement in NPI and FAB scores. The improvement seen in the tests was paralleled by the normalization of LICI and also by the enhancement of TMS-evoked EEG responses in the frontal lobes. 

The findings cited above come from small groups of patients. They still need replication and systematization, but they open a new perspective to investigate the functional aspects of neurodegenerative processes. One can also hope that such sensitive, neurophysiologic monitoring will contribute significantly to the optimization and individualization of current and future therapies. 

## 9. Limitations

The current study is not systematic and does not provide quantitative information. 

## 10. Conclusions

TMS offers new diagnostic and therapeutic possibilities in dementias. It is relatively cheap, non-invasive and safe and is continuously enriched with new modalities and paradigms of stimulation. TMS is a unique method to test cortical excitability and functionality of brain connections. For this reason, it may be regarded as a valuable adjuvant tool to other methods with confirmed efficacy in dementia such as amyloid positron emission tomography (amyloid-PET) [137] or assessment of levels of amyloid-beta1-42 and tau protein in cerebrospinal fluid [138]. TMS seems to be a good candidate for a primary diagnostic tool, especially for early screening for the presence of neurodegenerative processes, as it is in the case of MCI. The methods currently used in clinical settings, including the above-mentioned ones, may not be well-suited for such tasks. Amyloid-PET is expensive, and lumbar puncture is invasive and usually requires admission to the hospital. TMS may, therefore, help identify target patients in a timely way for new, experimental therapies, which usually require the inclusion of patients with relatively preserved cognitive functions. To date, the results of TMS research point out that the diagnostic approach, which includes all paradigms of cortical excitability, may be helpful to detect the signs of the neurodegenerative process and to differentiate between particular dementia types. Diagnostic specificity and sensitivity may be increased by implementing machine learning or by the recording of TMS-evoked potentials with electroencephalography. However, additional research is required to confirm these initial, promising findings. In the face of the absence of established disease-modifying therapies or pharmacological treatments with proven efficacy, rTMS may be one of the possible options for enhancing cognition. Multisite stimulation combined with training seems to enhance cognitive performance with particularly high efficacy, but systematic studies in dementias other than AD are still lacking. It seems that future research will focus on the development of multimodal diagnostic approaches including clinical, imaging and neurophysiological biomarkers, which will serve as a key to identifying high risk individuals for early therapeutic interventions.

## Data Availability

Not applicable.
